# Electroacupuncture and Rosiglitazone Combined Therapy as a Means of Treating Insulin Resistance and Type 2 Diabetes Mellitus: A Randomized Controlled Trial

**DOI:** 10.1155/2013/969824

**Published:** 2013-07-29

**Authors:** Rong-Tsung Lin, Huei-Chin Pai, Yu-Chen Lee, Chung-Yuh Tzeng, Chin-Hsien Chang, Pei-Hsiu Hung, Ying-I Chen, Tai-Hao Hsu, Chin-Chun Tsai, Jaung-Geng Lin, Shih-Liang Chang

**Affiliations:** ^1^Department of Internal Medicine and Emergency Medicine, Division of Endocrinology and Metabolism, Tungs' Taichung Metro Harbor Hospital, Taichung County, Taiwan; ^2^Department of Traditional Chinese Medicine, China Medical University Hospital, Taichung City, Taiwan; ^3^Department of Acupuncture, China Medical University Hospital, Taichung City, Taiwan; ^4^Department of Orthopedics, Taichung Veterans General Hospital, Taichung City, Taiwan; ^5^College of Life Science, National Tsing Hua University, Hsinchu City, Taiwan; ^6^Department of Traditional Chinese Medicine, En Chu Kong Hospital, Taipei, Taiwan; ^7^Division of Traditional Chinese Medicine, Chia-Yi Christian Hospital, Chiayi City, Taiwan; ^8^Department of Medicinal Botanicals and Health Applications, Da-Yeh University, Changhua County 51591, Taiwan; ^9^School of Chinese Medicine for Post-Baccalaureate, I-SHOU University, Kaohsiung County, Taiwan; ^10^School of Chinese Medicine, China Medical University, Taichung City, Taiwan

## Abstract

*Aims.* To evaluate the efficacy of rosiglitazone (TZD) and electroacupuncture (EA) combined therapy as a treatment for type 2 diabetes mellitus (T2DM) patients by randomized single-blind placebo controlled clinical trial. *Methods.* A total of 31 newly diagnostic T2DM patients, who fulfilled the study's eligibility criteria, were recruited. The individuals were randomly assigned into two groups, the control group (TZD, *N* = 15) and the experimental group (TZD + EA, *N* = 16). Changes in their plasma free fatty acid (FFA), glucose, and insulin levels, together with their homeostasis model assessment (HOMA) indices, were statistically compared before and after treatment. Hypoglycemic activity (%) was also compared between these two groups. *Results.* There was no significant difference in hypoglycemic activity between the TZD and TZD + EA group. The effectiveness of the combined therapy seems to derive from an improvement in insulin resistance and a significant lowering of the secreted insulin rather than the effect of TZD alone on T2DM. The combined treatment had no significant adverse effects. A lower plasma FFA concentration is likely to be the mechanism that causes this effect. *Conclusion.* This combined therapy seems to suppress endogenous insulin secretion by improving insulin resistance via a mechanism involving a reduction in plasma FFA. This trial is registered with ClinicalTrials.gov NCT01577095.

## 1. Introduction

Diabetes mellitus is a syndrome associated with a disordered metabolism and inappropriate hyperglycemia that can be due to either an absolute deficiency in insulin secretion or reduction in the biological effectiveness of insulin. Type 2 diabetes is the predominant form of diabetes worldwide and accounts for 90% of cases globally [1, 2]. Alarming increases in the prevalence of diabetes have occurred in Asia [3]. A number of factors have been shown to play an important role in the development of the disease and these include excessive caloric intake, a sedentary lifestyle, and abdominal visceral obesity [4]. In addition, many circulating inhibitors, including free fatty acids (FFAs), have also been found to be involved in reducing insulin sensitivity [5]. From a pathology point of view, patients with type 2 diabetes have a number of metabolic abnormalities including (1) resistance to the action of insulin in muscle, fat tissue, and liver; (2) defective insulin secretion, especially under glucose stimulus; and (3) increased glucose production by the liver [6].

Management of type 2 diabetes is a great challenge to physicians both because of the disease's complex pathology and because of the multiple chronic complications associated with the disease. The usual treatment for type 2 diabetes mellitus includes life style modification, exercise, diet therapy, oral antihyperglycemic drugs, and insulin. The thiazolidinediones represent a unique class of drug that may directly decrease insulin resistance by enhancing insulin action in skeletal muscle, liver, and adipose tissue [7]. Two of these compounds, rosiglitazone and pioglitazone, have been approved for clinical use in type 2 diabetic patients. In the UK Prospective Diabetes Study (UKPDS), reduction in the risk of microvascular complications (retinopathy, nephropathy, and neuropathy) was found in the intensive treatment group of patients with new onset type 2 diabetes [8]. However, it should be realized that there are possible unfavorable events associated with achieving lower glycemic targets such as hypoglycemia, bulky combinations of medications, and expense. Although present-day management of type 2 diabetes is more effective than before, as time goes by the effectiveness of drug treatment deteriorates in these patients. Novel therapies and different kinds of treatment for type 2 diabetes need to be developed in the future.

Acupuncture is a part of traditional Chinese medicine. According to traditional Chinese medicine theory, acupuncture regulates “Qi and Blood” and is likely to affect the bioavailability of substances taken internally and in the process may influence the absorption, distribution, metabolism, and/or excretion of substances [9]. In addition, researchers have begun using electroacupuncture (EA) rather than classical acupuncture. This approach combines traditional needle acupuncture with an electrical current passing through the needles into the acupoints. This seems to produce hypoglycemic responses, and, using EA at different frequencies, also causes the release of endogenous opioid peptides that activate specific receptors [10]. In this context, the potential of EA as a treatment for hyperinsulinemia is an important issue because type 2 DM may eventually develop into pancreatic failure [11].

Although the insulin sensitizer rosiglitazone improves insulin sensitivity, there are some adverse effects in terms of liver function and the induction of fluid retention. Since EA has been shown to improve insulin activity in our previous studies [12, 13], a combination of EA's effects in terms of enhancing insulin activity with the use of an insulin sensitizer may potentially be a new modality for the treatment of diabetes mellitus in humans [14]. In addition, this combined therapy may also improve insulin sensitivity and regulate the secretion of insulin, which ought to help to block any worsening of pancreas functioning.

The main purpose of this pilot study was to evaluate a combination of EA and drug therapy, namely, treatment with the insulin sensitizer, rosiglitazone, and to explore whether this improves insulin activity among type 2 diabetic patients. Therefore, we performed a clinical randomized controlled trial (RCT) on type 2 diabetic patients treated with rosiglitazone alone and compared these with patients treated with rosiglitazone combined with EA. The endpoints assessed were a reduction in blood glucose levels and/or improvements in insulin activity.

## 2. Materials and Methods

### 2.1. Study Design and Patient Population

We designed a randomized single-blind placebo controlled clinical trial to compare the reduction in blood glucose level and/or improvements in insulin resistance among type 2 diabetic patients who had been diagnosed within the last 5 years. These individuals were treated with rosiglitazone alone or with rosiglitazone combined with EA using a frequency of 15 Hz at the bilateral Zusanli acupoints. In addition, we studied possible factors associated with improvements in insulin resistance that might be associated with any synergism between EA and rosiglitazone. This was done by assaying plasma FFA, plasma insulin concentration, and calculating the HOMA index [15] for each group of patients. The study protocol was approved by the Institutional Review Board (IRB) of China Medical University Hospital in Taiwan and informed consent was requested and obtained from all study participants. These patients were recruited from the clinical endocrinology and metabolism section of internal medicine and the cooperative center of Chinese and Western Medicine, China Medical University Hospital in Taiwan.

### 2.2. Inclusion and Exclusion Criteria

We recruited patients who were eligible under the following criteria. The inclusion criteria were firstly that all were native Taiwanese patients aged from 20 to 65 years who had been diagnosed with type 2 diabetes mellitus within the last 5 years and who continued to use same antihyperglycemic agents to control their diabetes during the period of this study as previously. Secondly, their assessment was compatible with the diagnostic criteria of diabetes mellitus according to American Diabetes Association [16] in that (a) the patient presented with any one of the characteristic symptoms of diabetes mellitus such as thirst, polyuria, polyphagia, and weight loss with an any time single blood glucose estimation in excess of 11.1 mmol/L; (b) that the patient presented with a fasting blood glucose level in excess of 6.7 mmol/L after an overnight fast of 8 hours; and (c) that the patient had a 2-hour blood glucose estimation in excess of 11.1 mmol/L after a 75 g glucose load via an oral glucose tolerance test after an overnight fast of 8 hours.

The exclusion criteria were (1) individuals with nephrotic syndrome (urine protein over 3.5 g/day), edema or renal failure (serum creatinine over 115 *μ*mol/L); (2) individuals who had been diagnosed with heart failure (NYHA Fc III~IV) or who had had a pacemaker implanted; (3) individuals with abnormal liver function (GOT and GPT levels twofold above the normal range) or a diagnosis of liver cirrhosis; (4) individuals with a high HbA1C level (HbA1C above 9%); (5) pregnant women; (6) individuals who were receiving a thiazolidinedione class drug already; (7) individuals who were receiving insulin therapy already; (8) individuals who received an other therapy during the period of study; (9) individuals who were suffering from a homeostasis disorder or other systemic diseases; and (10) individuals who did not comply with the treatment during the study period.

### 2.3. Electroacupuncture

The EA treatment used the bilateral Zusanli acupoints, which are located on the anterior tibia muscle near the knees; these were identified based on previous studies [17]. After adjusting the EA apparatus to 15 Hz/10 mA (Han's Healthronics Likon, Taipei, Taiwan), 1.5-unit acupuncture needles (44 mm/32 gauge) were inserted 10–30 mm into the muscle layer at the selected acupoints. After needles were inserted to bilateral Zusanli acupoints, no specific manipulation was carried out; they were only inserted to the specific depth, which gave a sensation of *de-qi* and then electrical stimulation was begun. The positively charged (red pole) clip was connected to the right needle and the negatively charged (black pole) clip was connected to the left needle. In addition, in order to evaluate the effectiveness of the acupoint stimulating therapy and to avoid any variation that might influence the results, only one acupuncture doctor was invited to participate in this study.

### 2.4. Protocol

The patients enrolled in this clinical single-blind placebo controlled trial were distributed by permuted-block randomization. In each block, a random number for each treatment was generated on a calculator and this was used to decide the treatment group. Each patient was assigned to one of two groups according to this method of randomization by an independent research assistant. All patients were blinded to treatment assignment during the period of study. Body height, body weight, and body mass index were measured before treatment. Biochemical serum markers, namely, blood sugar, liver function, creatinine (Cr), HbA1C, lipid profile, insulin level, and FFA level were measured before treatment for each group. Blood sugar, insulin, and FFA levels were measured after treatment in order to allow an investigation of the efficacies of the protocols.

In the experimental (TZD + EA) group, enrolled patients were treated with 8 mg rosiglitazone 30 min before experiment after overnight fast for 8 hours. The TZD + EA group patients then received EA stimulation at the bilateral Zusanli acupoints with intensity of 15 Hz/10 mA for 30 min. Five mL of blood was drawn from cubital vein before and after EA stimulation for evaluation.

The placebo (TZD) group patients were treated with the same dose of rosiglitazone 30 min before experiment after an overnight fast for 8 hours. During the period of study, only electrode placement took place at the bilateral Zusanli acupoint with no acupuncture manipulation or EA stimulation; this acted as the placebo treatment. Similar to above, five mL of blood was drawn from cubital vein before and after placebo treatment for evaluation.

### 2.5. Laboratory Measurements

Plasma glucose (mmol/L) concentrations were determined using a spectrophotometric system (COBAS System, Roche Diagnostics Ltd., Rotkreuz, Switzerland) and commercially available enzymatic kits run in duplicate. The plasma FFA levels (meq/L) were determined spectrophotometrically on the COBAS System (Roche Diagnostics) using a commercially available nonesterified fatty acid kit (Randox Laboratories Ltd, Ardmore, United Kingdom). The plasma insulin levels were measured using an ELISA kit (Linco Research, St. Charles, MO, USA) [18]. In brief, samples were incubated for 2 hours at room temperature in a shaker and exposed to peroxidase conjugate and antibodies bound to a 96-well plate. The conjugate acted on the 3,3′,5,5′-tetramethylbenzidine. The reaction was stopped by adding 1 M sulfuric acid 100 *μ*L and the mixture was shaken to produce a colorimetric endpoint, which was measured spectrophotometrically. The values obtained were in international unit of peptide per liter of plasma.

### 2.6. Outcome Measures

The primary endpoint was plasma glucose level after treatment in each group patients and a comparison of the hypoglycemic activity between the two independent groups. The secondary endpoints were a comparison of the changes in plasma insulin level, HOMA index, and FFA level after treatment for each group of patients together with a comparison of the FFA level percent change between the two independent groups. All measurements were performed by one independent technician who was blinded to the treatment assignments of the patients throughout the study and the side effects of all the treatments were also recorded.

### 2.7. Statistical Analysis

The hypoglycemic activity (%) was calculated as follows: (Gi−Gt) × (100/Gi); where Gi is the initial glucose concentration and Gt is the concentration after treatment. All values were expressed as means ± SEM in the figures and tables. The Wilcoxon signed-rank test was applied to assess differences by treatment for each group of dependent samples. The Mann-Whitney test was applied to compare the differences between two independent groups. For all comparisons, a *P* value less than 0.05 (two-sided) was considered statistically significant.

## 3. Results

### 3.1. Baseline Data

**Figure 1:**
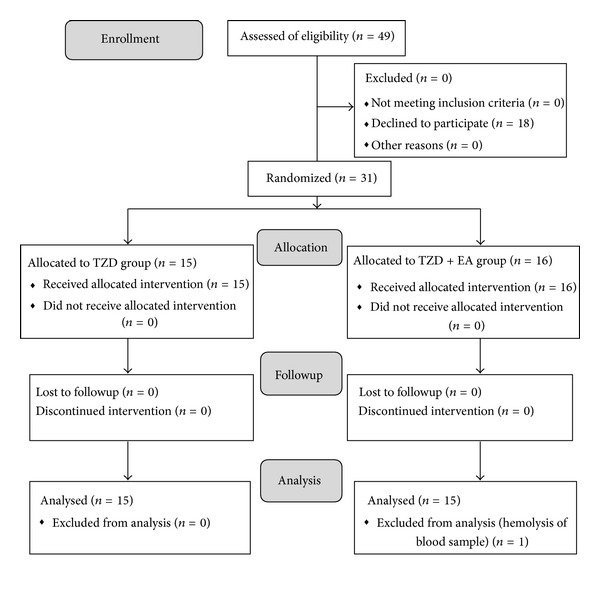
The flowchart diagram of progress through the various phases of this two-arm randomized trial according to CONSORT, which stands for consolidated standard for reporting trials.

In this study, 49 patients were recruited and 31 patients completed the clinical protocol with one blood sample being excluded from analysis because of hemolysis. The flowchart of the interventions is shown in Figure 1. All patients were recruited from April, 2006 to May, 2007 at China Medical University Hospital, Taichung, Taiwan and this trial was stopped by a prior setting protocol. A total of 18 patients declined to participate in this study after it was explained to them by the researcher. The male : female ratios were 10 : 6 for the TZD + EA group and 8 : 7 for the TZD group. The baseline characteristics of the intension-to-treat patients in the two groups are shown in Table 1. No statistical difference at baseline was observed among these two groups by non-parametric Mann-Whitney test. In addition, cases were followed by telephone to identify any relevant adverse effects after completion of this study. No obvious side effects were observed during this study except for one case where a localized wheal at an acupoint occurred due to scratching after EA treatment. Antihistamine ointment was prescribed for this and the skin recovered well; the patient was able to tolerate further therapy during the trial after this treatment.

### 3.2. Effect of Combined Therapy on Plasma Glucose Level

After one treatment of each group, a trend in hypoglycemic response was noted for both groups, but a significant hypoglycemic effect was only noted in the TZD group (7.7 ± 0.6 to 7.4 ± 0.6 mmol/L, *P* < 0.05) by Wilcoxon signed-rank test (Table 2). Furthermore, there was no significant difference in hypoglycemic activity (%) between the combined therapy (EA + TZD, *n* = 15) and monotherapy (TZD, *n* = 15) groups after treatment by Mann-Whitney test (Table 2).

### 3.3. Effect of Combined Therapy on Plasma Insulin Level and HOMA Index

**Figure 2:**
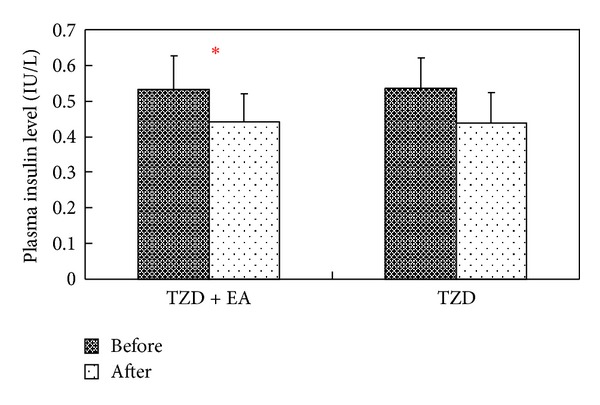
Effects of combined therapy or single therapy on plasma insulin concentration among type 2 diabetic patients before and after treatment. TZD + EA = patients receiving 8 mg rosiglitazone stat and electroacupuncture; TZD = patients receiving 8 mg rosiglitazone stat only; Wilcoxon signed-rank test was used to assess differences in the means of the groups, *P < 0.05.

**Figure 3:**
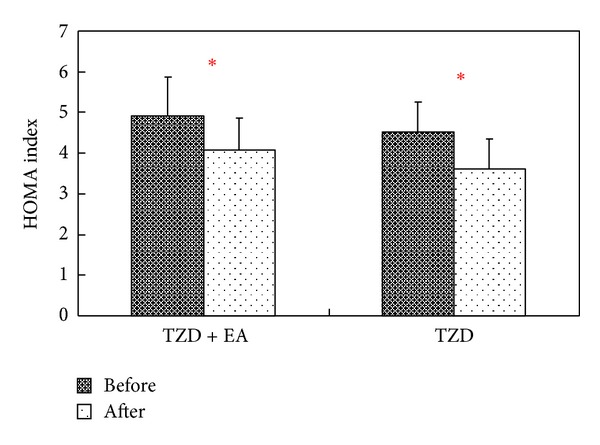
Effects of combined therapy or single therapy on insulin sensitivity as evaluated by HOMA index among type 2 diabetic patients before and after treatment. TZD + EA = patients receiving 8 mg rosiglitazone stat and electroacupuncture; TZD = patients receiving 8 mg rosiglitazone stat only; HOMA index = (fasting plasma glucose × fasting plasma insulin)/22.5; Wilcoxon signed-rank test was used to assess differences in the means of each group, *P < 0.05.

A significant reduction in plasma insulin level was noted after treatment only in the TZD + EA group (*P* < 0.05) by Wilcoxon signed-rank test (Figure 2). Simultaneously, the HOMA indices before and after treatment were calculated in order to evaluate insulin resistance within the combined therapy and TZD groups. The results showed that the HOMA index after treatment was significantly lower than before treatment for both groups (*P* < 0.05) by Wilcoxon signed-rank test (Figure 3).

### 3.4. Effect of Combined Therapy on Plasma FFA Level

**Figure 4:**
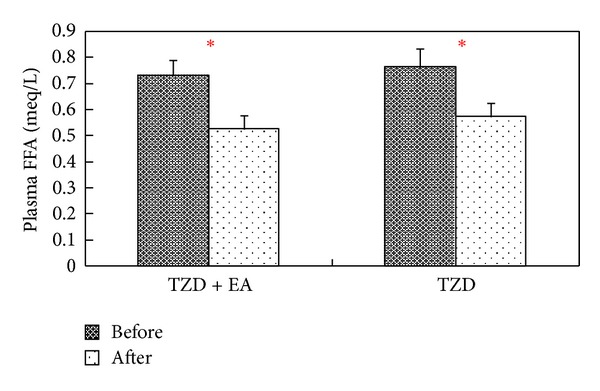
Effect of combined therapy or single therapy on plasma FFA concentration among type 2 diabetic patients before and after treatment. TZD + EA = patients receiving 8 mg rosiglitazone stat and electroacupuncture; TZD = patients receiving 8 mg rosiglitazone stat only; Wilcoxon signed-rank test was used to assess differences in the means of each group, *P < 0.01.

The plasma FFA levels were significantly decreased for both groups (*P* < 0.05) by Wilcoxon signed-rank test (Figure 4). Nevertheless, no significant difference in plasma FFA lowering activity (%) was observed between the two groups by Mann-Whitney test.

## 4. Discussion

Although clinical studies have demonstrated that acupuncture is useful because it can lower plasma glucose levels [19], most studies have focused on its effects with respect to diabetic neuropathy [20]. The commonly used acupoints for DM like syndromes include Pishu (BL20), Geshu (BL17), Zhongwan (CV12), Sanyinjiao (SP6), Neiguan (PC6), and Zusanli (ST36) [21]. In this study, we have carried out the first randomized clinical trial that compares the effect of combined therapy (rosiglitazone + EA) on insulin resistance in type 2 diabetic patients with rosiglitazone therapy alone. This design was based on the previous animal studies, which have shown that EA stimulation at the bilateral Zusanli acupoints produced a greater hypoglycemic response than EA stimulation at Zhongwan acupoints, and that 15 Hz EA is able to produce a better hypoglycemic effect than 2 Hz EA on the same acupoints [17, 22]. On another hand, according to the animal studies, the hypoglycemic activity was obtained after 30-min EA, and rosiglitazone was taken orally 30 min before the patient entered into this experiment to allow absorption and metabolism of drug. This is the reason why the blood was taken 30 min after EA treatment. Since the metabolic abnormalities of type 2 diabetes are complex, more than one medication is often needed for the majority of patients over time to allow maintenance of plasma glucose levels at as near-normal range as possible in order to reduce microvascular complications [23]. At present, the thiazolidinediones are approved for use in combination with metformin, sulfonylureas, glinides, and insulin [24]. Therefore, the combination with EA is possible an alternative.

In the present study, no significant difference in hypoglycemic activity between the patients in the combined therapy group and the patients in the TZD group was identified; nonetheless a trend towards a lowering of plasma glucose levels was found for the patients in both groups. These results are different from our previous animal studies, which showed that the plasma glucose lowering activity of rosiglitazone was increased by EA in both normal and neonatal STZ-induced type 2 diabetic rats [14]. A number of conditions were different between the present study and the earlier animal studies, particularly in which the animal studies were carried out under general anesthesia, while the human subjects were fully conscious during the clinical trial. One possibility is that the plasma glucose lowering effect in patients from the combined treatment group may have been masked by environmental factors, such as pain and stress during EA. Another possibility is that there may have been selection bias because a higher percentage of male patients were excluded because they were already being prescribed thiazolidinedione class drugs.

There were some other limitations that affected this study. We chose an inclusion for subjects with a hemoglobin A1C below 9%; however, it was not possible to determine whether this was appropriate. The results may have been different if we had recruited patients with HbA1C values between 7% and 10%. Furthermore, although no significant hypoglycemic effect was detected when the combined treatment group was compared with the single drug treatment group, this result may have been related to the relatively short period of treatment. Finally, the sample size in the present study was small and any significant differences between the two groups may have been masked by this. The sample size was also limited by a disappointing recruitment rate, and the protocol was terminated by the researchers based on the available circumstances, and because the prior guarantee period of the protocol from the IRB had ended. Both of these events resulted in the sample size being smaller than that of the prior setting. As a result of these limitations, nonparametric statistical methods were applied to evaluate the differences between the two groups and between before and after treatment.

The normal secretion of insulin is essential to the maintenance of normal glucose tolerance, and an abnormally high secretion of insulin is a consistent finding among type 2 diabetic patients. Usually, insulin resistance and hyperinsulinemia are the main clinical causes of type 2 diabetic among patients, and these indicate the presence of an impaired biological response to either exogenously administered or endogenously secreted insulin [25]. When there is insulin resistance, beta cells compensate for the insulin resistance by increasing insulin secretion even in the presence of normal glucose concentrations. Compensatory hyperinsulinemia due to insulin resistance reflects a combination of increased insulin production and decreased insulin clearance, but most evidence seems to suggest that increased insulin secretion is the predominant factor [26]. In this study, the patients in both the combined treatment group and the single TZD treatment group exhibited a lower HOMA index after treatment. Interestingly, the combined therapy was able to significantly reduce the higher levels of insulin secretion found in these type 2 diabetic patients, which will result in a meaningful relief in terms of pancreas stress that may lead to pancreas failure. That is to say, this combined therapy seems to have the potential benefit of extending the time before pancreatic beta cell failure occurs with type 2 diabetic patients rather than improving insulin resistance. This result is compatible with our previous studies that have shown that insulin sensitivity is enhanced by EA stimulation at the bilateral Zusanli acupoints of normal male Wistar rats and of streptozotocin induced diabetic rats when evaluated by the HOMA index [12, 13]. Furthermore, in another study on the effect of EA on insulin sensitivity among steroid background male Wistar rats, the EA group rats were found to also exhibit lower HOMA indices compared to the non-EA group rats [27]. It has been proposed that this effect occurs via the enhancement of endogenous insulin activity by EA. Since abnormal hyperinsulinemia has been found to diminish insulin sensitivity, elevated serum levels of insulin may cause insulin resistance by downregulating insulin receptors and desensitizing postreceptor pathways [28]. Other studies also showed that 24 hours and 72 hours of sustained physiological hyperinsulinemia in a normal individuals are able to specifically inhibit the ability of insulin to increase nonoxidative glucose disposal and that this is associated with an impaired ability of insulin to stimulate glycogen synthase activity [29]. Our results are similar to previous studies showing that suppression of insulin secretion in insulin resistant individuals results in increased insulin sensitivity [30]. This suggests that a combination therapy consisting of one-dose of rosiglitazone and EA together is able to suppress endogenous insulin secretion, which in turn improves insulin activity as evaluated by the HOMA index. Thus, we conclude that EA stimulation at a specific acupoint may have an important potential role for the treatment of type 2 diabetes because this treatment is able to enhance insulin activity.

Although the combined therapy was able to enhance insulin activity by suppressing endogenous insulin secretion in this study, biochemical evidence for the mechanistic basis of EA activity in this context needs to be explored. Previous studies have shown that 15 Hz EA at the bilateral Zusanli acupoints is able to enhance insulin activity by decreasing plasma FFA concentration and upregulating the expression of insulin signal proteins in steroid background male Wistar rats [27]. Another study has shown that the mechanism by which FFA induces insulin resistance involves the intramyocellular and intrahepatocellular accumulation of triglycerides and diacylglycerol, which then reduce tyrosine phosphorylation of insulin receptor substrate-1 (IRS-1) and IRS-2 [31]. In addition, studies have shown that EA (15 Hz) at the Zusanli acupoint treatment is able to induce a hypoglycemic response in streptozotocin induced diabetic rats by stimulating the cholinergic nerves, which in turn stimulate the expression of insulin signaling proteins [32]. Additionally, the action of EA has a combined effect that involves both cholinergic nerve stimulation and increased nNOS activity via a lowering of the plasma FFA concentration; the result is enhanced glucose tolerance as described in our recently published reports [13, 27, 33, 34]. Taking the above findings as a whole, we were therefore interested in the role of FFA and how it influences insulin activity when diabetic patients are treated using the combination therapy.

FFA is one of the key factors that influences insulin activity and elevated FFA levels are predictive of the progression from impaired glucose tolerance to diabetes [35]. The majority (>80%) of patients with early T2DM in the USA are overweight [36] and characterized by long-term elevations in their plasma FFA concentration. These are not suppressed as normally they occur following ingestion of a mixed meal [37] or in response to insulin [38]. In addition, insulin is a potent inhibitor of lipolysis and suppresses the release of FFA from the adipocyte by inhibiting the enzyme hormone-sensitive lipase [31]. In our study, a significant plasma FFA lowering effect was noted with both groups of patients. These results are compatible with other studies showing that in early type 2 diabetics, the ability of insulin to inhibit lipolysis and to reduce the plasma FFA concentration is markedly impaired [31]. In addition, it has been found that progressively elevated plasma FFA concentrations seem to induce gluconeogenesis and eventually compensatory hyperinsulinemia; these may then lead to insulin resistance in muscle and liver. This contrasts with a decrease in plasma FFA concentration, which is able to inhibit glucose-stimulated insulin secretion [32]. Thus, in addition to the documented analgesic effect of EA, we have shown in this study that there is a possible systemic effect of EA whereby there is modulation of FFA levels that can, in turn, influence insulin secretion in patients with type 2 diabetes.

In conclusion, the combined therapy of electroacupuncture and rosiglitazone seems to be able to suppress endogenous insulin secretion, which then improves insulin activity as evaluated by the HOMA index. This seems to occur through a reduction in plasma FFA levels and does not seem to have any major side effects among patients with type 2 DM. Therefore, in the near future, a long-term and large sample size assessment of combined therapy is needed to evaluate the possible benefits that this approach may have in terms of improving the risk profiles of type 2 DM patients.

## Figures and Tables

**Table 1:** Baseline data obtained from the intention-to-treat patients in the experimental (TZD + EA) and placebo groups (TZD).

Groups	TZD + EA (*N* = 16)	TZD (*N* = 15)	*P*-Value
Age (Years)	48 ± 2	50 ± 2	0.74 (NS)
Body Height (cm)	167 ± 2	165 ± 3	0.27 (NS)
Body Weight (Kg)	73 ± 3	69 ± 3	0.54 (NS)
BMI (Kg/m^2^)	26 ± 1	25 ± 1	0.93 (NS)
Glucose (mmol/L)	9.4 ± 0.5	9.1 ± 0.6	0.51 (NS)
HbA1C (%)	8.10 ± 0.45	8.15 ± 0.44	1.00 (NS)
Cr (*μ*mol/L)	71.6 ± 2.7	69.8 ± 5.3	0.20 (NS)
GPT (IU/L)	31 ± 4	28 ± 3	0.71 (NS)
Cholesterol (mmol/L)	5.1 ± 0.3	4.9 ± 0.3	0.44 (NS)
Triglyceride (mmol/L)	1.6 ± 0.3	1.6 ± 0.3	0.81 (NS)
Take OHA (Yes : No)	10 : 6	10 : 5	0.91 (NS)

All values are shown as mean ± SEM except for the use of oral hypoglycemic agents (OHA); TZD + EA = experimental group, who were patients that received 8 mg rosiglitazone and electroacupuncture; TZD = placebo group, who were patients that received 8 mg rosiglitazone only. Independent groups were compared using the nonparametric Mann-Whitney test. NS, means no significant difference, *P* > 0.05.

**Table 2:** Effects of combined and single-dose TZD therapy on plasma glucose.

Group	Before treatment	After treatment	Hypoglycemic activity (%)
TZD + EA (*N* = 15)	8.4 ± 0.4	8.2 ± 0.5	−2 ± 2
TZD (*N* = 15)	7.7 ± 0.6	7.4 ± 0.6*	−5 ± 2

Plasma glucose concentrations are presented as mean ± SEM (mmol/L), *N* = number of patients; the Wilcoxon signed-rank test was used to assess differences in the means of the plasma glucose concentration of each group before and after treatment; *P < 0.05. The non-parametric Mann-Whitney test was used to compare the difference in hypoglycemic activity between the two independent groups, *P* > 0.05.

## Abbreviations

EA: Electroacupuncture
TZD: Rosiglitazone
T2DM: Type 2 diabetes mellitus
FFA: Free fatty acid
HOMA: Homeostasis model assessment index.

## References

[1] Alberti KG, Zimmet PZ (1998). Definition, diagnosis and classification of diabetes mellitus and its complications. Part 1: diagnosis and classification of diabetes mellitus provisional report of a WHO consultation. *Diabetic Medicine*.

[2] Zimmet P, Alberti KGMM, Shaw J (2001). Global and societal implications of the diabetes epidemic. *Nature*.

[3] Yoon KH, Lee JH, Kim JW (2006). Epidemic obesity and type 2 diabetes in Asia. *Lancet*.

[4] Goodyear LJ, Kahn BB (1998). Exercise, glucose transport, and insulin sensitivity. *Annual Review of Medicine*.

[5] Hunter SJ, Garvey WT (1998). Insulin action and insulin resistance: diseases involving defects in insulin receptors, signal transduction, and the glucose transport effector system. *American Journal of Medicine*.

[6] Ostenson CG (2001). The pathophysiology of type 2 diabetes mellitus: an overview. *Acta Physiologica Scandinavica*.

[7] Olefsky JM, Saltiel AR (2000). PPAR*γ* and the treatment of insulin resistance. *Trends in Endocrinology and Metabolism*.

[8] Turner R (1998). Intensive blood-glucose control with sulphonylureas or insulin compared with conventional treatment and risk of complications in patients with type 2 diabetes (UKPDS 33). *Lancet*.

[9] Senna-Fernandes V, França D, Moreno SFR (2006). The effect of "Zusanli" (ST. 36) acupuncture on the bio-availability of sodium pertechnetate in Wister rats. *Acupuncture and Electro-Therapeutics Research*.

[10] Lin JG, Chen WC, Hsieh CL (2004). Multiple sources of endogenous opioid peptide involved in the hypoglycemic response to 15 Hz electroacupuncture at the Zhongwan acupoint in rats. *Neuroscience Letters*.

[11] Nolan CJ (2010). Failure of islet *β*-cell compensation for insulin resistance causes type 2 diabetes: what causes non-alcoholic fatty liver disease and non-alcoholic steatohepatitis?. *Journal of Gastroenterology and Hepatology*.

[12] Chang SL, Lin KJ, Lin RT, Hung PH, Lin JG, Cheng JT (2006). Enhanced insulin sensitivity using electroacupuncture on bilateral Zusanli acupoints (ST 36) in rats. *Life Sciences*.

[13] Lee YC, Li TM, Tzeng CY (2011). Electroacupuncture-induced cholinergic nerve activation enhances the hypoglycemic effect of exogenous insulin in a rat model of streptozotocin-induced diabetes. *Experimental Diabetes Research*.

[14] Pai HC, Tzeng CY, Lee YC (2009). Increase in Plasma Glucose Lowering Action of Rosiglitazone by Electroacupuncture at Bilateral Zusanli Acupoints (ST.36) in Rats. *JAMS Journal of Acupuncture and Meridian Studies*.

[15] Matthews DR, Hosker JP, Rudenski AS (1985). Homeostasis model assessment: insulin resistance and *β*-cell function from fasting plasma glucose and insulin concentrations in man. *Diabetologia*.

[16] American Diabetes Association (1998). Screening for type 2 diabetes. *Diabetes Care*.

[17] Chang SL, Lin JG, Hsieh CL, Cheng JT (2002). Comparision of hypoglycemic effect in different acupoints response to 2 Hz electroacupuncture. *The Journal of Chinese Medicine*.

[18] Porstmann T, Kiessig ST (1992). Enzyme immunoassay techniques. An overview. *Journal of Immunological Methods*.

[19] Chen D, Gong D, Zhai Y (1994). Clinical and experimental studies in treating diabetes mellitus by acupuncture. *Journal of Traditional Chinese Medicine*.

[20] Wei RX, Xiang J, Dong Q (2007). Considerations on syndrome differentiation and acupuncture treatment of diabetic peripheral neuropathy. *Journal of Acupuncture and Tuina Science*.

[21] Wang Q (2003). The present situation of TCM treatment for diabetes and its researches. *Journal of Traditional Chinese Medicine*.

[22] Chang SL, Tsai CC, Lin JG, Hsieh CL, Lin RT, Cheng JT (2005). Involvement of serotonin in the hypoglycemic response to 2 Hz electroacupuncture of zusanli acupoint (ST36) in rats. *Neuroscience Letters*.

[23] Ohkubo Y, Kishikawa H, Araki E (1995). Intensive insulin therapy prevents the progression of diabetic microvascular complications in Japanese patients with non-insulin-dependent diabetes mellitus: a randomized prospective 6-year study. *Diabetes Research and Clinical Practice*.

[24] Nathan DM, Buse JB, Davidson MB (2009). Medical management of hyperglycaemia in type 2 diabetes mellitus: a consensus algorithm for the initiation and adjustment of therapy: aonsensus statement from the American Diabetes Association and the European Association for the Study of Diabetes. *Diabetologia*.

[25] Kahn R (1998). Consensus development conference on insulin resistance: 5-6 november 1997. *Diabetes Care*.

[26] Jones CNO, Pei D, Staris P, Polonsky KS, Chen YDI, Reaven GM (1997). Alterations in the glucose-stimulated insulin secretory dose-response curve and in insulin clearance in nondiabetic insulin-resistant individuals. *Journal of Clinical Endocrinology and Metabolism*.

[27] Lin RT, Tzeng CY, Lee YC (2009). Acute effect of electroacupuncture at the Zusanli acupoints on decreasing insulin resistance as shown by lowering plasma free fatty acid levels in steroid-background male rats. *BMC Complementary and Alternative Medicine*.

[28] Olefsky JM, Revers RR, Prince M (1985). Insulin resistance in non-insulin dependent (type II) and insulin dependent (type I) diabetes mellitus. *Advances in Experimental Medicine and Biology*.

[29] Del Prato S, Leonetti F, Simonson DC, Sheehan P, Matsuda M, DeFronzo RA (1994). Effect of sustained physiologic hyperinsulinaemia and hyperglycaemia on insulin secretion and insulin sensitivity in man. *Diabetologia*.

[30] Alemzadeh R, Langley G, Upchurch L, Smith P, Slonim AE (1998). Beneficial effect of diazoxide in obese hyperinsulinemic adults. *Journal of Clinical Endocrinology and Metabolism*.

[31] Groop LC, Bonadonna RC, DelPrato S (1989). Glucose and free fatty acid metabolism in non-insulin-dependent diabetes mellitus. Evidence for multiple sites of insulin resistance. *Journal of Clinical Investigation*.

[32] Denis McGarry J (2002). Dysregulation of fatty acid metabolism in the etiology of type 2 diabetes. *Diabetes*.

[33] Lin RT, Chen CY, Tzeng CY (2011). Electroacupuncture improves glucose tolerance through cholinergic nerve and nitric oxide synthase effects in rats. *Neuroscience Letters*.

[34] Chang SL, Lee YC, Li TM (2011). Electroacupuncture at the Zusanli (ST-36) acupoint induces a hypoglycemic effect by stimulating the cholinergic nerve in a rat model of streptozotocine-induced insulin-dependent diabetes mellitus. *Evidence-Based Complementary and Alternative Medicine*.

[35] Charles MA, Eschwège E, Thibult N (1997). The role of non-esterified fatty acids in the deterioration of glucose tolerance in Caucasian subjects: results of the Paris prospective study. *Diabetologia*.

[36] Mokdad AH, Ford ES, Bowman BA (2000). Diabetes trends in the U.S.: 1990–1998. *Diabetes Care*.

[37] Reaven GM, Hollenbeck C, Jeng CY, Wu MS, Chen YDI (1988). Measurement of plasma glucose, free fatty acid, lactate, and insulin for 24 h in patients with NIDDM. *Diabetes*.

[38] Boden G, Shulman GI (2002). Free fatty acids in obesity and type 2 diabetes: defining their role in the development of insulin resistance and *β*-cell dysfunction. *European Journal of Clinical Investigation*.

